# Nano-scale mechanisms explain the stiffening and strengthening of ligament tissue with increasing strain rate

**DOI:** 10.1038/s41598-018-21786-z

**Published:** 2018-02-27

**Authors:** Angelo Karunaratne, Simin Li, Anthony M. J. Bull

**Affiliations:** 1 Department of Bioengineering, Imperial College London, London, SW7 2AZ UK; 2 Present Address: Department of Mechanical Engineering, University of Moratuwa, Moratuwa, Sri Lanka; 3 Present Address: Wolfson School of Mechanical, Electrical and Manufacturing Engineering, Loughborough University, Loughborough, UK

## Abstract

Ligament failure is a major societal burden causing disability and pain. Failure is caused by trauma at high loading rates. At the macroscopic level increasing strain rates cause an increase in failure stress and modulus, but the mechanism for this strain rate dependency is not known. Here we investigate the nano scale mechanical property changes of human ligament using mechanical testing combined with synchrotron X-ray diffraction. With increasing strain rate, we observe a significant increase in fibril modulus and a reduction of fibril to tissue strain ratio, revealing that tissue-level stiffening is mainly due to the stiffening of collagen fibrils. Further, we show that the reduction in fibril deformation at higher strain rates is due to reduced molecular strain and fibrillar gaps, and is associated with rapid disruption of matrix-fibril bonding. This reduction in number of interfibrillar cross-links explains the changes in fibril strain; this is verified through computational modelling.

## Introduction

Ligaments provide passive restraint to shear forces at joints. Thus ligament injuries can lead to osteoarthritis^1^, joint instability and pain^2^. Mechanical properties and deformation and failure mechanisms of ligaments have mostly been studied at relatively low loading rates whereby an increasing strain rate up to 1/s causes Young’s modulus and failure stress to increase and strain-to-failure to decrease^3^. However, these loading rates are not representative of those seen in trauma, and there is currently no knowledge of the underlying mechanisms at the micro- and nanoscale.

Collagen type I is the most abundant structural protein in ligaments, where it determines deformation and failure mechanisms^4^. Type I collagen fibrils are held together by carbohydrate cross-links (proteoglycans (PGs) and glycoproteins)^5^. At the ångström scale, tropocollagen molecules are tightly bound by these carbohydrate cross links and enzymatic and non-enzymatic crosslinks to make collagen fibrils with 65–67 nm axial D-periodicity. Mesoscopic fascicles thus have a crimping pattern and aggregate to form the ligament^6^. The mutual relationship among these elements at different hierarchical levels during high strain rate loading as seen in trauma is not known.

The macroscopic stress-strain curve of ligaments exhibits three distinct regions: toe, heel and linear^7^. Each region is associated with simultaneously-occurring mechanisms at different length scales: tropocollagen molecule extension (mode 1); change in periodicity (mode 2); and sliding between collagen fibrils (mode 3) that is controlled by the interspersed proteoglycan matrix^8^. Initially, a small load is adequate to elongate the tissue, due to microscopic uncrimping (toe region). Subsequently, sequential straightening of disordered molecular kinks in collagen fibril gaps leads to rapid tissue stiffening (heel region). Then, collagen triple helices’ elongation and molecule gliding initiates the curve’s linear portion.

Although significant efforts have been made to characterise these deformation mechanisms, and their relationship to hierarchical structure, at low strain rates^7,9^, ligament ruptures are associated with much higher strain rates. For example, strain rates during normal walking are estimated to be in the range of 0.001–1/s^10^, during downhill running in the range of 0.1–1/s^11^ and even higher in blast trauma. Other studies have examined tissue level ligament material properties at physiologically realistic strain rates, and have provided valuable information on bulk mechanical properties, but they have not been able to explain the structure-function relationship of individual components in the hierarchically-structured material of ligaments.

A fundamental understanding of scale-dependent mechanics at physiologically-relevant strain rates will allow us to understand, simulate, and predict ligament injuries. This information will also help to identify the optimum properties that are required for biomaterials and scaffolds to promote organisation, growth and differentiation of cells in the process of forming engineered functional tissues.

Here we investigate deformation and failure mechanisms at the nanoscale by measuring deformations of individual components of collagen fibrils at physiological strain rates. We use a nano mechanical imaging technique - *in-situ* synchrotron small angle X-ray diffraction (SAXD) – in uni-axial tensile testing across strain rates to examine the deformation and failure mechanisms of human knee ligament tissue at multiple length scales. Based on our experimental results we propose a model to explain failure mechanisms of collagen fibrils at the nano-scale, implement this model in a finite element simulation, and validate the model against experimental observations.

## Results

**Figure 1:**
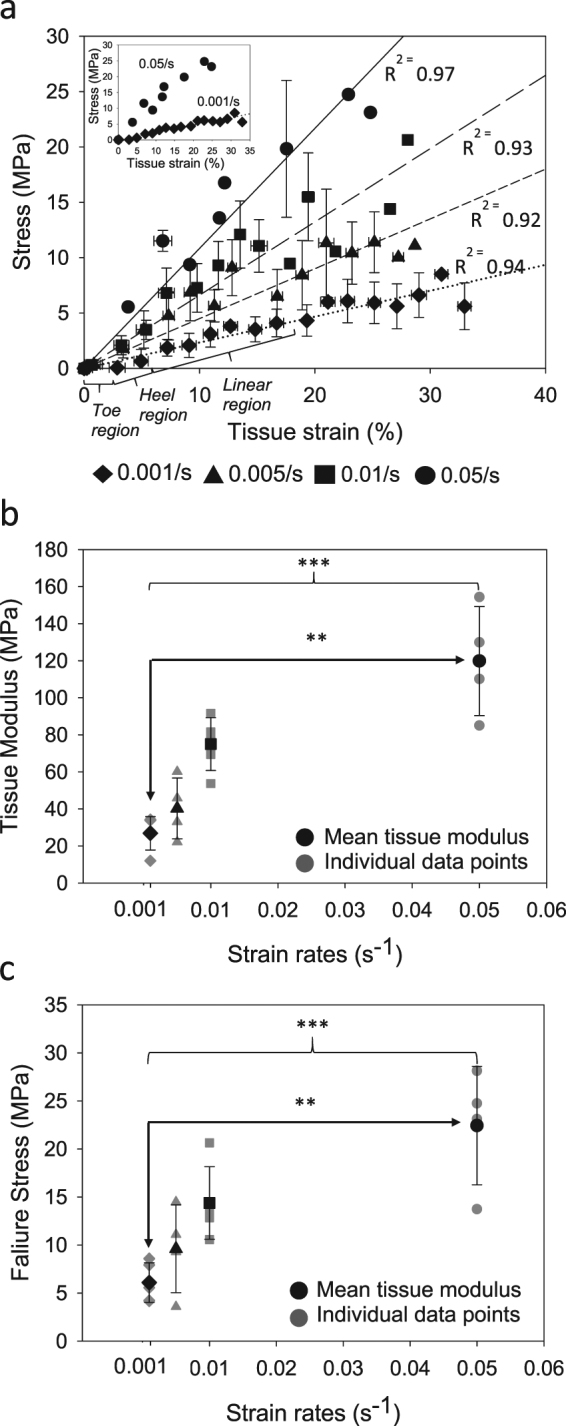
Tissue level ligament properties (**a**) Stress vs. measured tissue strain % (n = 5 for each strain rate, error bars are SD). Lines are linear regressions. Insert showing stress vs. measured tissue strain % for the slowest and fastest strain rates tested in this study. (**b**) Tissue modulus (individual data points and mean ± SD for n = 5) (**c**) Failure stress (individual data points and mean ± SD for n = 5). (*p < 0.05, **p < 0.01, ***p < 0.001.)

Our results showed that the shape of the stress-strain curve at the tissue level changes dramatically from slow (toe, heel and linear) to fast (linear only) strain rates as strain rate is increased (Fig. 1a and insert). The ligament elastic modulus (Fig. 1b) and failure stress (Fig. 1c) were increased by 346.7% (p = 0.001) and 268.3% (p = 0.001), respectively, from slowest to fastest strain rates. This strain rate dependent behaviour is in agreement with previous studies on ligaments and tendons^12,13^.

**Figure 2:**
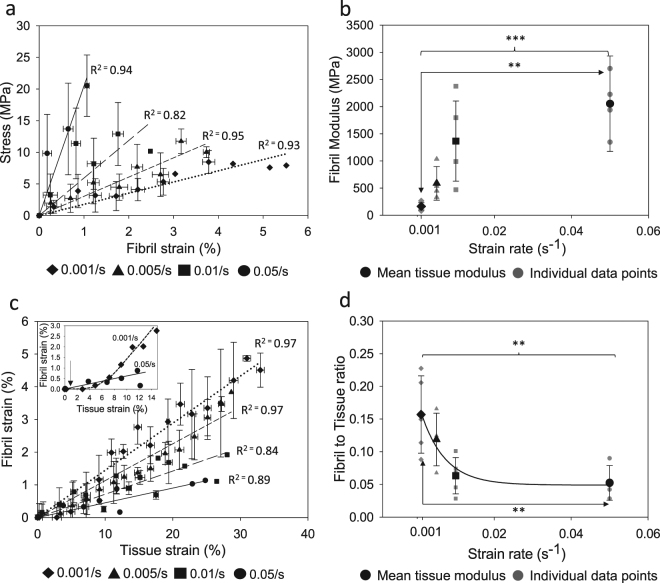
Fibril level ligament properties (**a**) Stress vs. fibril strain % (n = 5 for each strain rate, error bars are SD). Lines are linear regressions. (**b**) Fibril modulus (individual data points and means ± SD for n = 5) (**c**) Fibril vs. tissue strain (n = 5 for each strain rate, error bars are SD). Lines are linear regressions. Insert showing fibril vs. tissue strain data shown for the fastest and slowest strain rates tested in this study. The arrow is pointing to the toe region of the slow strain rate curve. (**d**) Fibril to tissue strain ratio (individual data points and mean ± SD for n = 5). (*p < 0.05, **p < 0.01, ***p < 0.001.)

At the nanoscale level the fibril modulus (slope of stress vs fibril strain; Fig. 2a) increases at high strain rates (by 1158.7% from 0.001/s to 0.05/s, p = 0.00023; Fig. 2b). Fibril strain as a function of tissue strain is shown in Fig. 2c. At low strain rates, fibrillar strain increases linearly with applied tissue strain after passing through the initial toe and heel regions up to 4.5% of maximum fibril strain (Fig. 2c insert) in the elastic regime. No heel or toe regions were observed at high strain rates. Beyond the heel region, the strain in collagen fibrils increases much slower in higher strain rates compared to lower strain rates (p = 0.00505). The linear region of the fibril to tissue strain ratio that was calculated (Fig. 2d) decreases exponentially with the strain rate and no further reduction was observed beyond 0.01/s.

**Figure 3:**
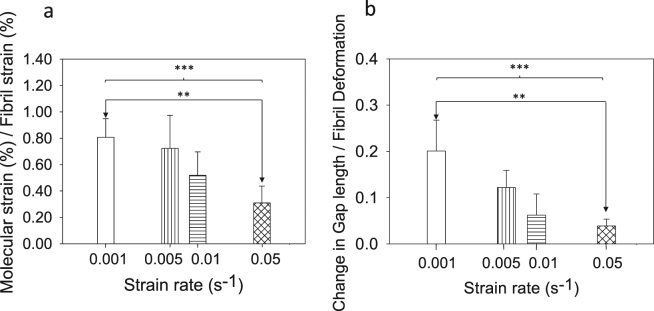
(**a**) Mean molecular strain % to fibril strain % ratio and (**b**) change in gap length to fibril deformation. Error bars are standard deviations (n = 5 for each bar). (*p < 0.05, **p < 0.01, ***p < 0.001.)

The ratios of molecular strain to fibril strain (Fig. 3a) and the ratios of change in gap length to fibril deformation (Fig. 3b) reveal that at lower strain rates (0.001/s and 0.005/s) the major contribution to the collagen deformation comprises molecular elongation (0.83 ± 0.36 and 0.72 ± 0.25) and change in gap length (0.20 ± 0.07 and 0.12 ± 0.04); these contributions were significantly reduced at higher strain rates (molecular deformation by 52%, p = 0.00503, and change in gap length by 81%, p = 0.0041). These experimental results led us to hypothesize that rapid loading/unloading leads to breakage of cross-links between collagen fibrils, thus promoting inter-fibrillar slippage/gliding and damage prior to macroscopic failure.

**Figure 4:**
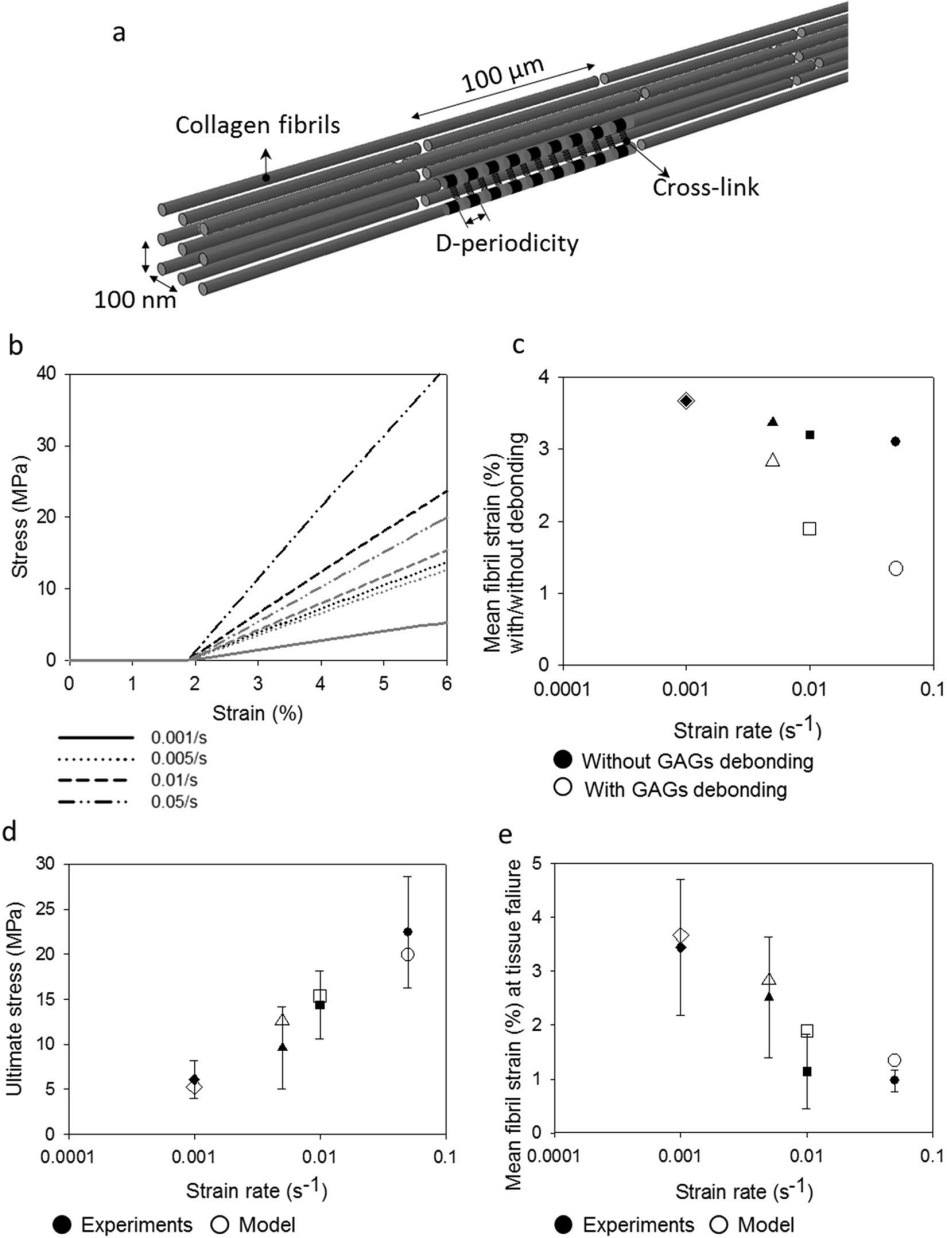
Computational model verification. (**a**) Finite Element model of fibril networks: individual fibrils consist of overlap (black) and Gap (grey) regions of tropocollagen fibrils, interconnected with neighbouring collagen fibrils through cross-links (black springs). Due to periodicity, a unit cell of two individual fibrils were modelled. (**b**–**e**) Finite element modelling results of the strain rate dependent mechanical behaviour of ligament tissue: (**b**) stress-strain curves of ligament tissue with (grey dash lines) and without (black dash lines) cross-links debonding; (**c**) mean fibril strain with (hollowed) and without (filled) cross-links debonding for both overlaps and gap regions of collagen fibrils at different strain rates; comparing (**d**) ultimate stress and (**e**) mean fibril strain at different strain rates. Error bars are standard deviations for the experimental data only (n = 5 for each bar).

To test the hypothesis, we developed a finite element model consisting of repeating unit cells (Fig. 4a) of collagen fibrils with inter-fibrillar cross-links (GAGs) attaching to adjacent fibrils at every D-period. The model was initially verified against models in the literature at a quasi-static strain rate^14,15^. Subsequently, a strain rate dependent viscoelastic behaviour of collagen fibrils was added to the model (using our experimental data in Fig. 2) to evaluate the effect of strain rate dependent fibril behaviour on tissue stiffness, and the interfibrillar deformation mechanism with and without a cross-link debonding mechanism (Fig. 4b,c). The model confirms that stiffening of collagen fibrils contributes considerably to the increase in overall tissue modulus at higher strain rates (Fig. 4b–e). Further, the results obtained from our parametric study of the interfibrillar cross-link deformation mechanism reveal that the number of cross-links between adjacent fibrils has a significant effect on the value of average fibril strain. As the strain rate increases, the number of effective interfibrillar cross-links reduces considerably, from full activation at quasi-static strain rate to only 5%, equivalent to 75 cross-links, at 0.01/s.

## Discussion

Our tissue level mechanical property characterisation has revealed that tissue modulus (Fig. 1b) and failure stress (Fig. 1c) increase from quasi static to higher strain rates, in good agreement with previous studies. The toe, heel and linear regions were reproduced at low strain rates (Fig. 1a inset). At higher strain rates, toe and heel regions were not observed, and instead there was only a relatively small linear region followed by a rapid macroscopic fracture. In order to elucidate the mechanisms governing our tissue results at high strain rates, we sought to investigate fibrillar and molecular level kinetics at quasi static and physiologically relevant strain rates.

Using small angle scattering, we measured collagen fibrillar level deformation at low and high strain rates. At low strain rates and below 5% applied tissue strain, no fibril deformation was observed. This is in good agreement with results reported in an atomic force microscopy functional imaging study on tendons^16^. This behaviour corresponds to loss of collagen fibre crimping at the microscopic level^17^. As suggested in the literature, we found that, during inelastic deformation, individual fibrils move independently within the fibres they constitute^6^. Our experimental results provide evidence that the overall tissue strain in the stretched ligament is larger than the strain in the individual fibrils, indicating that relative movements within the matrix are taking place. If loading continues, some of the fibrils may begin to fail, therefore damage may accumulate and lead to ultimate macroscopic failure. Collagen fibres are composites, comprising stiff collagen fibrils embedded in a soft matrix of hydrated PGs in which GAG sidechains of PGs bind to specific regions along the collagen fibrils. These interconnectors (predominantly representing GAGs, along with other interweaving fine elastin, fibrils and fibrillin), are believed to act mostly as shear load transmitters from one fibril to its neighbouring fibrils^14,18,19^ in the collagen network^13,14^. At low strain rates, these cross-links facilitate inter-fibrillar sliding and protect individual collagen fibrils from over strain in the inelastic regime. This inelastic deformation allows the ligament to stretch further at higher length scales without inducing complete failure of the fibrils.

**Figure 5:**
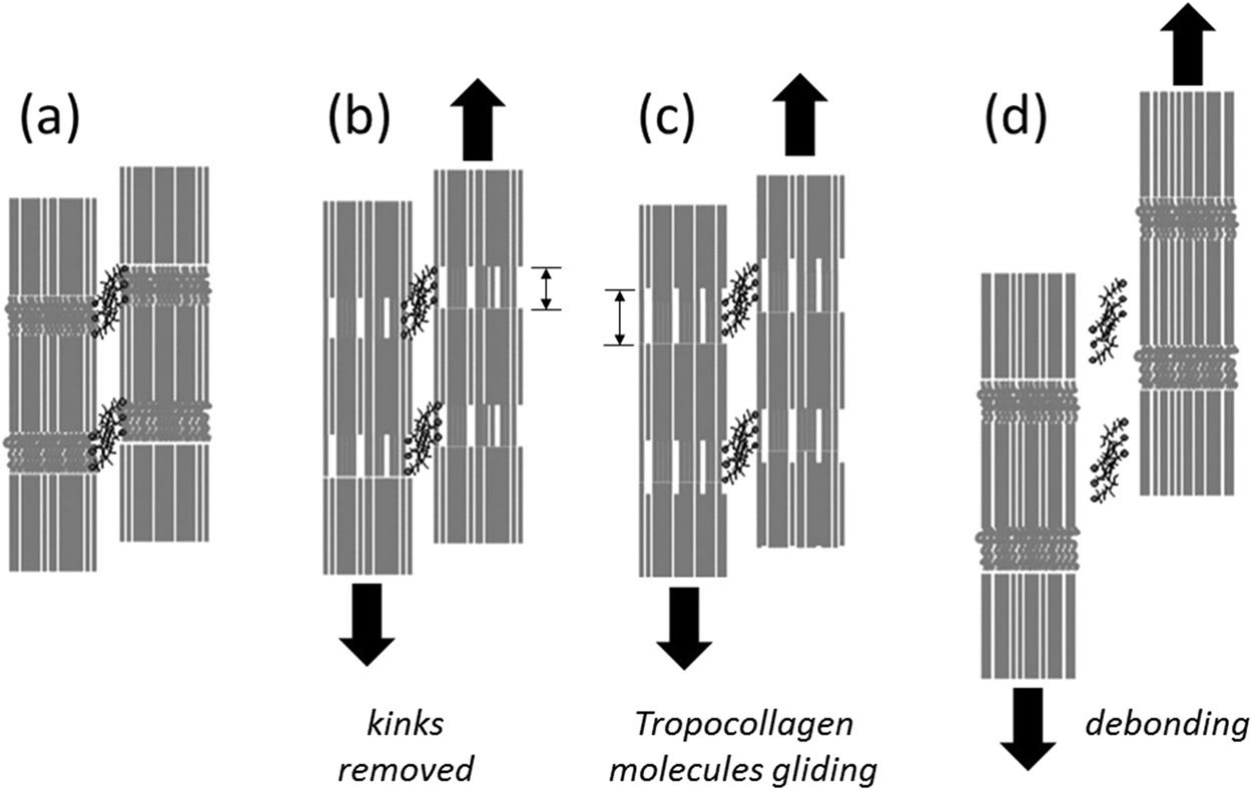
Proposed fibrillar level deformation and failure model as a schematic. One unit cell consists of two fibrils connected with GAGs side chains (black mesh). Each fibril is made up of a collection of staggeredly oriented tropocollagen molecules (grey individual lines). (**a**) Unloaded state. (**b**) Toe region - Removed kinks in the gap regions of collagen fibrils. (**c**) Linear region - Tropocollagen molecules starts to glide past each other. (**d**) Debonded stiff matrix from collagen fibrils.

At higher strain rates, individual ligament collagen fibrils exhibit higher stiffness (Fig. 2a,b) and lower strain (Fig. 2c) compared to slow strain rates. This contradicts experimental studies performed on tendon tissues^9^. Low fibril to tissue ratio at higher strain rates implies that, for an applied tissue strain within the linear region, less deformation occurs within the fibril. The origin of this stiffening of the collagen fibrils at higher strain rates is found at a lower level of the tissue hierarchy. In contrast to quasi static strain rates (0.001/s and 0.005/s), fibril deformation was observed from the outset when relatively fast strain rates were applied to the tissue (Fig. 2c). This behaviour is an indication of the absence of the heel region in the stress-tissue strain curve at higher strain rates, which implies that sequential straightening of disordered molecular kinks in the gap region of the collagen fibrils does not occur. This is confirmed by our results showing that the change in gap length to fibril deformation ratio significantly decreases with increasing strain rates (Fig. 3b). Therefore, at higher strain rates, we suggest that the most probable mechanism of deformation is stretching of tropocollagen molecules and the cross-links between the helices, which explains the steep linear region in the stress-fibril strain curve (Fig. 2a). The rapid loading experienced in higher strain rates limits fibrillar deformation only up to 2%, whereas fibrils stretch up to 5% before failure when slow strain rates are applied (Fig. 2c). This implies that even though tropocollagen molecules start to stretch during high strain rates, this molecular deformation process does not persist, and failure mechanisms prevail. Our experimental results show a reduction in molecular strain to fibril strain ratio at higher strain rates (Fig. 3a), which provides an explanation for reduced fibril deformation. In the context of a fibrillar deformation mechanism, if contributions from molecular stretching and change in gap length decrease at higher strain rates, the contribution from fibrillar sliding has to have a relative increase. We hypothesize that, during high strain rate loading, part of the interfibrillar sidechains become detached or inactive from the collagen fibrils while the remaining cross-links and other inactive substances^9^ continue to provide support to transfer load from one fibril to another, until macroscopic failure is reached, when load drops to zero instantaneously. Figure 5 illustrates this proposed model as a schematic. These strain rate sensitive mechanisms avoid excessive deformation of collagen fibrils and elevate the ligament tissue modulus at higher strain rates.

The time-dependent deformation mechanism of interfibrillar cross-links proposed in this study was simulated computationally to further understand the functional connection between collagen fibrils and interfibrillar cross-links. An initial simulation was carried out using a unit cell consisting of two collagen fibrils connected fully with cross-links (Fig. 4a), which over predicts tissue stress at higher strain rates due to collagen fibril stiffening and under predicts the reduction of fibrillar deformation. This means that the viscous stiffening effect alone of collagen fibrils cannot cause the reduction in fibril strain at higher strain rates; there could be additional strain rate sensitive load shielding mechanisms that act at an inter-fibril level to mitigate deformation of collagen fibrils at higher strain rates. In order to test this proposed mechanism, fibril breakage from the matrix was modelled by deactivating interfibrillar cross-links at higher strain rates, which promotes the free gliding of collagen fibrils. With this modification, there is a good agreement between the model and the experimental results. We did not introduce failure mechanisms in our model, so we caution against over interpretation of these findings. Nevertheless, this parametric study further confirms our hypothesis that collagen-fibril load sharing is dependent upon a matrix crosslinking mechanism. The rapid disruption of matrix-fibril bonding would lead to less stress transmitted via crosslinks. This in turn would promote fibrillar sliding, resulting in reduced fibrillar strain at higher strain rates.

The cooperative functioning of hierarchical structural elements in biological fibrous tissues like ligaments and tendons has long been identified as crucial for their physiological function^3,9,20^. However, little is known about deformation and failure mechanisms at different length scales during physiologically relevant strain rates. Therefore in this study, simultaneous *in-situ* tensile testing and synchrotron X-ray diffraction experiments were performed on human lateral collateral ligament tissue to investigate the deformation and failure mechanisms at rates from physiological loading through to traumatic injurious events. These multiscale (from Å to mm scale) experimental results demonstrate that ligament tissues are strain rate sensitive at all length scales and allow us to develop a new hypothesis on failure mechanism of fibrous tissue at physiological strain rates. To test this hypothesis, an FE model of collagen fibrous network with and without strain rate dependent cross-links debonding/activation was proposed. The results of our model suggest that, in addition to the strain rate dependent fibril stiffening, the number of active cross-links also reduces considerably at higher strain rates. This results in an increase in the number of debonded cross-links and leads to a reduction of average fibril strain, promoting interfibrillar slippage.

In conclusion, synchrotron X-ray diffraction, mechanical testing and computational modelling was used to investigate and explain the nano scale mechanical property changes of ligament tissue across strain rates. We found that tissue-level stiffening is mainly due to the stiffening of collagen fibrils and the reduction in fibril deformation at higher strain rates is due to reduced molecular strain and fibrillar gaps, and is associated with rapid disruption of matrix-fibril bonding.

## Materials and Methods

### Materials

Tissue samples were provided by the Imperial College Healthcare NHS Trust Tissue Bank (ICHTB) that is supported by the National Institute for Health Research (NIHR) Biomedical Research Centre based at Imperial College Healthcare NHS Trust and Imperial College London. The views expressed are those of the author(s) and not necessarily those of the NHS, the NIHR or the Department of Health. All methods were carried out in accordance with relevant guidelines and regulations. This study was approved by Imperial College Research Ethics Committee and informed consent was provided by donor relatives.

The ligament tissue from a left knee lateral collateral ligament (LCL) of a 48 year old male was used. Initially the LCL of the knee joint was isolated by removing skin and muscle. Following harvesting, the middle 30 mm section of the specimen was isolated by removing the posterior and distal sections of the ligament through blunt dissection to ensure that ligament fibrils were not disrupted. Blunt dissection was then again used to separate the specimen in equal width strips of 3 mm in width and depth. These were cut down into twenty 10 mm long strips. These strips were randomly allocated to four groups each of five samples.

### Sample preparation

For mechanical testing, both ends of the ligament tissue strips were secured in a custom holder and then embedded in water-resistant dental cement (Filtek^TM^ Supreme XT, 3 M ESPE, USA). These embedded ends were used to grip the specimen in the micro mechanical testing machine^21^ with full submersion in phosphate buffered solution (pH 7.4) to simulate physiological conditions. Embedded specimens were further trimmed in the transverse direction using blunt dissection in line with the fascicles. This created a test specimen with a long thin middle section (4 mm gauge length, 2 mm in thickness and 3 mm in width) of a relatively constant cross sectional area. Quick setting stiff alginate paste was used as a cast to measure mid-substance cross sectional area for later calculation of engineering stress. This technique has been previously used to measure cross sectional area of ligament tissues^3^.

### *In situ* tensile testing with small angle X-ray diffraction (SAXD)

#### Experimental setup

The SAXD combined with mechanical testing was performed at the high brilliance I22 beamline at Diamond Light Source (Oxfordshire, United Kingdom). The ligament specimens were mounted into a previously described custom designed micro tensile rig with video extensometry for tissue deformation detection^22,23^. Time-resolved X-ray diffraction patterns were taken simultaneously, at intervals dependent on the strain rate used for each specimen. The testing machine was mounted on a 3-axis motorized stage in the I22 beamline so that the longitudinal direction of the collagen fibres was perpendicular to the incoming X-ray beam. Load parameters were measured with a tension/compression load cell (SLC31/00005; RDP Electronics, West Midlands, UK) during tensile testing. Tissue strain was measured by tracking the deformation of ink markers on the specimen surface, using video extensometry. These marks were 1 mm vertically apart, each 1 mm away from the clamped ends. The ligament specimens were kept partially immersed in phosphate buffered saline during the X-ray experiments. Temperature was maintained between 18–21 °C for the duration of testing.

#### Micro and nano level investigation

Twenty human lateral collateral ligament strips were used in this study to measure fibrillar deformation at 0.001, 0.005, 0.01, and 0.05/s (5 specimens for each strain rate). The fastest strain rate used in this study was limited by the maximum acquisition rate of the SAXD detector. Each specimen was stretched until a tare load of 0.05 N was detected. In order to align the specimen in the testing machine, five pre tensing cycles were performed from 0.05 N to 1 N and then brought back to 0.05 N; gauge length (distance between ink markers) at this load was considered as the initial specimen length for tissue strain calculations. The first SAXD image was collected at this 0.05 N load value and used to calculate D periodicity at the unstretched length. Each specimen was stretched to failure, with SAXD spectra (1 s exposure time) taken for every 1% applied tissue strain. The D-periodicity (~ 67 nm) of the meridional banding patterns in the collagen fibrils arising from the intrafibrillar tropocollagen packing was tracked to calculate fibril strain while recording tissue strain and load^21,22^. A synchrotron X-ray beam with wavelength 0.886 Å and a beam cross section 10 × 12 *µ*m was used to measure the SAXD patterns. SAXD patterns were collected using a 2 M Pilatus detector system which was positioned 1 m away from the specimen as measured with a calibration standard (type I collagen from rat tail tendon). Each SAXD data frame had a pixel resolution of 2048 × 2048 pixels and a pixel size of 172 × 172 *µ*m^2^.

#### Molecular level investigation

Deformation of collagenous tissue has 3 modes at the micro, nano and molecular levels. In an attempt to understand strain rate sensitivity at the molecular level rearranging modes (modes 2 and 3) or in other words changes in O/D, we used relative intensity of reflections corresponding to the D-period.

Electron density in the gap region – *ρ*_0_

Electron density in the overlap region – *ρ*_0_ + *Δρ*

Therefore the relative intensity of *n*^th^ order reflection is as follows (^24^; Equation 1)

1 $${I}_{n}(s)=K{(\Delta \rho )}^{2}[\sin (n\pi [O/D])/(n\pi )[O/D]]$$

For the above equation, when the lattice factor and *K* become constant, change in O/D can be determined by calculating the ratio of the relative intensity for the *(n* + *1)*^th^ order reflection peak to that of the *n*^th^ one as shown elsewhere (^24^; Equation 2).

2 $${I}_{n+1}/{I}_{n}={(n/(n+1))}^{2}[\frac{[\sin ((n+1)\pi [O/D])]}{\sin (n\pi [O/D])}]$$

In this study, intensities of third and fifth reflection peaks were obtained from SAXD patterns to determine change in O/D ratios for each tissue strain measurement until failure. The determined O/D values are comparable with those obtained in a previous study^24,25^. Subsequently the change in gap region and the molecular strain were calculated using O/D for each strain rate as described previously^24^.

### Finite element analysis

To further elucidate the nano-scale deformation mechanisms and to test the hypothesis derived from experimental results on the ultrastructural constituents of ligament tissue at different strain rates, a finite element model was developed to simulate the mechanical behaviour of the collagen fibrous network at the nano-scale. The use of molecular dynamics simulation requires very high strain rates to be applied to the model, usually in the range of shock loading, and was outside the physiological strain rate and length scale (micro-nano) of this study. The developed model considers the collagen fibrous network as a three phase structure consisting of (i) overlap and (ii) gap regions of collagen fibrils alternating at every 67 nm interval (D-period) with (iii) interfibrillar cross-links attaching to adjacent fibrils at every D-period. The spatial arrangement of the collagen fibrous network was adapted from previous studies^14,15^. Each fibril was modelled as a slender beam structure staggered halfway to its neighbouring fibrils (100 nm distance) in a tetragonal packed assembly (Fig. 4a). The length, diameter and interfibrillar spacing of individual collagen fibrils were 100 µm, 180 nm and 100 nm, respectively^14,15^. A unit cell consisting of two adjacent collagen fibrils was considered in the analysis due to the periodic arrangement of fibrils.

Collagen fibrils were modelled as consisting of strain-rate dependent viscoelastic material based on the experimental results from the current study and results in the literature^6,26^. This assumes that the average elastic modulus between the overlap and gap regions (assuming that the overlap region is 20% stiffer than the gap region^27^,) of collagen fibrils follows a bilinear^3^ relationship (Equation 3):

3 $${E}_{avg}={A}_{1}(\dot{\varepsilon })+{C}_{1}\,{if}\,(\dot{\varepsilon } < {\dot{\varepsilon }}_{0});{E}_{avg}={A}_{2}(\dot{\varepsilon }-{\dot{\varepsilon }}_{0})+{C}_{2}\,{if}\,(\dot{\varepsilon } > {\dot{\varepsilon }}_{0});{C}_{2}={A}_{1}({\dot{\varepsilon }}_{0})+{C}_{1}$$

where *A*_*i*_, *C*_*i*_ (*i* = 1, 2) and $\dot{\varepsilon }$ are material parameters based on best fit to the experimental results (*R*^2^ = 0.7786), and *E*_*avg*_ and $\dot{\varepsilon }$ are the mean elastic modulus of collagen fibrils and strain rate, respectively (Table 1). The exact mechanism of GAGs transferring load is debated^28^ and so we modelled the interfibrillar deformation mechanism as discrete cross-links, representing not only GaGs, but also transverse/interweaving fibrils and fine elastin fibres, which are thought to provide transverse and shear load support^29^, as bilinear spring elements^14,15^ with: (i) low initial stiffness *k*_*cl*_ ≈ 0 between 0% to 800% nominal strain, and (ii) high stiffness *k*_*cl*_ = 5^.^3 × 10^−11^ nN/nm when nominal strain is above 800%, following previous studies^14,15^. The collagen fibril network was stretched uniaxially at the strain rates used in the physical experiments (0.001 s^−1^, 0.005 s^−1^, 0.01 s^−1^ and 0.05 s^−1^) up to 6% of tensile strain. A user subroutine (USDFLD) was employed to define the strain rate dependent elastic moduli of the overlap and gap regions of collagen fibrils.

**Table 1:** Strain rate dependent material parameters of mean elastic modulus of collagen fibrils fitted using obtained experimental data.

A_1_ (MPa∙s)	A_2_ (MPa∙s)	C1 (MPa)	${\dot{\varepsilon }}_{0}$(s^−1^)
99490	22880	131.74	0.01019

The debonding effect of the cross-links was modelled by assigning a close to zero stiffness to the cross-links which were modelled as nonlinear spring elements. The number of active cross-links was varied from 50 (1/30 active cross-links) to 1492 (full cross-links) between two individual fibrils and the results were optimized against the experiment by minimizing the sum of squares of the relative errors for both tissue stress and average fibril strain.

### Statistical analysis

All statistical analyses were performed using SPSS (Version 22.0, IBM Corp., Armonk, NY). Measurements are reported as mean ± s.d. Differences between the four strain rates were tested using one way ANOVA and, where significance was seen, Tukey’s HSD post-hoc test was used to identify where the differences lay. The significance threshold was chosen as p = 0.05. In figures error bars are standard deviations.
